# Techno-Environmental Evaluation of Alkaline Treatment in Flax Reinforced Thermoplastics

**DOI:** 10.3390/polym16050662

**Published:** 2024-02-29

**Authors:** Suhail Hyder Vattathurvalappil, Mian Mobeen Shaukat, Rajesh Theravalappil, Shahzada Zaman Shuja, Wael Gamaleldin Abdelrahman

**Affiliations:** 1 Department of Aerospace Engineering, King Fahd University of Petroleum and Minerals, Dhahran 31261, Saudi Arabia; wgamal@kfupm.edu.sa; 2 Interdisciplinary Research Center for Advanced Materials, King Fahd University of Petroleum and Minerals, Dhahran 31261, Saudi Arabia; 3 Department of Mechanical Engineering, King Fahd University of Petroleum and Minerals, Dhahran 31261, Saudi Arabia; mshaukat@kfupm.edu.sa (M.M.S.); shuja@kfupm.edu.sa (S.Z.S.); 4 Interdisciplinary Research Center for Sustainable Energy Systems, King Fahd University of Petroleum and Minerals, Dhahran 31261, Saudi Arabia; 5 Interdisciplinary Research Center for Refining and Advanced Chemicals, King Fahd University of Petroleum and Minerals, Dhahran 31261, Saudi Arabia; rajesh@kfupm.edu.sa; 6 Interdisciplinary Research Center for Aviation and Space Exploration, King Fahd University of Petroleum and Minerals, Dhahran 31261, Saudi Arabia

**Keywords:** flax fibers, high density polyethylene, life cycle assessment, mechanical properties, environmental impact

## Abstract

A combination of thermoplastics and natural fiber reinforcements is considered an ideal choice to mitigate environmental impacts and enhance recyclability or reusability. Chemical treatments are often employed to enhance the thermomechanical properties of natural fiber-reinforced plastics. Nevertheless, it is of paramount importance to assess the techno-economic impact of such chemical treatments and environmentally friendly materials for their implementation in mass productions on an industrial scale. In this work, high-density polyethylene is reinforced with sodium hydroxide (NaOH)-treated and untreated flax fibers to study its impact on mechanical and environmental properties. The composites treated with NaOH exhibited a 37% increase in tensile strength. However, life cycle assessment performed on the NaOH-treated samples showed that they had a global warming potential of 5.8 kg of CO_2_, a terrestrial acidification potential of 0.0269 kg of SO_2_, and a human carcinogenic toxicity of 0.031 kg of 1,4-DCB compared to the untreated samples. In summary, the techno-environmental analysis reveals a novel approach to identifying chemical treatments based on their technical and environmental effects.

## 1. Introduction

Flax fibers are increasingly being used as reinforcement in thermoplastics because of their remarkable mechanical properties and their sustainable, biodegradable, reversible, non-abrasive, and ecologically advantageous characteristics. Thermoplastics have reversible characteristics, whereas flax exhibits adaptability, cost-effectiveness, wide availability, and equivalent strength to glass fibers. These fibers, which are obtained from the inner bark of the stem of cellulosic, multi-cellular bast fiber, are typically 10–100 cm in length, 40 to 80 μm in diameter, and are stronger than cotton fibers as their polymers lie almost parallel to the fiber axis. Flax fibers are commercially utilized in a variety of configurations. They can be used as monofilament reinforcement, manufactured mats, yarns, rovings, or fabrics [1,2].

High density poly-ethylene (HDPE) is widely applied in piping, container, and other utility applications owing to its UV and chemical resistance. A recent study conducted by Diwahar et al. showed that HDPE reinforced with flax fibers can effectively be recycled to fabricate other ecofriendly alternatives [3]. Moreover, the study confirmed the added mechanical characteristics improvements of adding natural fiber layers to HDPE, including tensile strength, flexural strength, impact strength, and hardness. However, flax fibers undergo a series of chemical treatments that enhance their capability for adhesion with polymers, resist moisture, and increase their longevity. Scanning electron microscopy (SEM) was used to study the fiber surface changes due to chemical treatments. Although micrographs revealed an overall decrease in fiber diameter, they also confirmed a reduction in grooves with increased duration of chemical treatment, exhibiting uniformity along the fiber surface by reducing the variation of the fiber diameter along its length. Some of the chemicals used in the treatments are sodium hydroxide (NaOH), peroxide, silane, maleic anhydride, potassium permanganate, and acetic anhydride [4,5,6,7,8,9,10,11,12]. These change the flax fiber’s physical, chemical, and adhesion properties. In these studies, the increase in tensile strength and single fiber pull-out stress varies with the type of treatment. For example, treatment with stearic acid yields superior values of these mechanical properties in comparison to potassium permanganate. Moreover, it was found that melaic anhydride grafted PP (MAPP) staple fibers improve the mechanical properties of natural fiber thermoplastic composites. The higher melt flow rate combined with lower share viscosity results in composites of superior modulus and strength compared to those manufactured from flax/PP blend. NaOH is frequently employed as a preliminary treatment prior to the application of other chemical treatments. In addition, treating flax fibers with NaOH makes it easier remove impurities and make the surface rougher, which results in better mechanical interlocking, and improves their overall performance. Even though these treatments have benefits in terms of chemical and thermomechanical properties, they can lead to high production costs and emissions into the environment.

To meet the growing threat of climate change, it is essential to examine the environmental impacts of new processes and materials, and reduce greenhouse gas emissions and energy consumption in every stage of manufacturing processes. In fact, in the manufacturing sector, production of raw materials is responsible for a large percentage of global environmental footprint. Reducing the environmental impacts of raw materials is therefore very important to meet net zero emissions targets. Normally, the environmental impacts of new processes and materials developed in a lab are not studied in detail until they are ready for industrial production. At that stage it is often difficult to change the processes or other factors of production to reduce the environmental impacts. It is therefore important to study the environmental impacts of processes to develop novel materials at an early stage of development. Investigating the environmental impacts of these processes, however, can help to mitigate their environmental impacts. The method used to measure the environmental impacts of flax fiber composites for this study is life cycle assessment (LCA).

Life cycle assessment (LCA) is one of the most widely used standardized methodologies for determining environmental impacts of products, processes, and systems. Initial LCA studies were conducted to compare environmental impacts of various products in the industry. However, over the years the method has been standardized and has gained a wide spread acceptance as a tool to quantify environmental impacts. For about the last thirty years, it has been widely used in industrial and research settings to evaluate the sustainability of processes and systems [13]. The method is governed in the ISO standards [14] and is widely reported in the scientific literature. LCA takes into account all the stages involved in production processes. It then helps to identify the environmental impacts of various stages of the life cycle of a product including material extraction, transportation, manufacturing, recycling, etc. LCA helps to identify environmental hotspots—the stages that create the most damaging environmental impacts—in the life cycle of a product or a process. This in turn helps to guide the activities that can lead to overall reduction of environmental footprints. LCA also helps to prevent environmental burden shifting from one life cycle stage to the other stage. For example, using a novel and lightweight material in a vehicle may reduce the environmental impact of the use stage by reducing the amount of fuel needed to run the vehicle, but this material may cause harmful emissions during recycling, offsetting the gains in the use phase. Several researchers have used LCA to highlight negative environmental impacts. For example, Rubin [15] has used it to determine the environmental impacts of recycling circuit boards. In this study, LCA will be used to determine the environmental impacts of flax-reinforced thermoplastics [16]. This will help to highlight potential drivers of these impacts.

This study involves the evaluation of untreated and alkaline-treated flax fiber-reinforced HDPE composites, focusing on their technical and environmental aspects. The tensile properties of the laminated sandwich construction were analyzed to investigate the effects of alkaline treatment. In addition, a life cycle assessment was performed on both untreated and alkaline-treated samples in order to compare and analyze the resulting advantages.

## 2. Experimental Details

### 2.1. Materials

The composites were manufactured using unidirectional flax strands and high-density polyethylene. Uni-directional flax fiber mats were obtained from Vruksha Composites, an Indian company (Tenali, India). The maker employed a weaving process to create a unidirectional fabric mat ensuring that the flax fibers were evenly spaced and positioned at consistent intervals. SABIC provided high-density polyethylene pellets of the P6006AD grade.

### 2.2. Alkaline Treatment and Composite Manufacturing

A 10% solution of NaOH was used to prevent the deterioration of flax fiber mats and facilitate fibrillation. A 10% solution of NaOH was produced by dissolving 10 g of NaOH in a 100-mL volumetric flask using distilled water. The flax mats were immersed in a 10% NaOH solution for 3 h at room temperature. It underwent cleansing using distilled water and was then neutralized using a solution of acetic acid (glacial, ≥99.85%) at a ratio of 10 mL per 1 L of water. Subsequently, the mats underwent multiple rinses with new distilled water until complete removal of NaOH was achieved. Following this, the mats were dried in an oven set at a constant temperature of 80 °C for a duration of 5 h. The desiccated mats were kept in an airtight plastic bag.

The process of creating a laminated sandwich composite of flax-reinforced HDPE via compression molding involved two distinct phases. The HPDE pellets underwent compression molding in order to create the facial skin during step 1. During this procedure, high-density polyethylene (HDPE) pellets were inserted into a hollow steel mold and subjected to a pressure of 100 bar and a temperature of 200 °C. A twenty-second degassing process was inserted in the middle of the compression molding at zero pressure. This procedure eliminated the entrapped air bubbles within the composite material. Subsequently, the materials were subjected to cooling under the same pressure for a duration of 12 min until their temperature hit 30 °C. The size of the plates manufactured were 160 mm × 160 mm × 1 mm.

**Figure 1:**
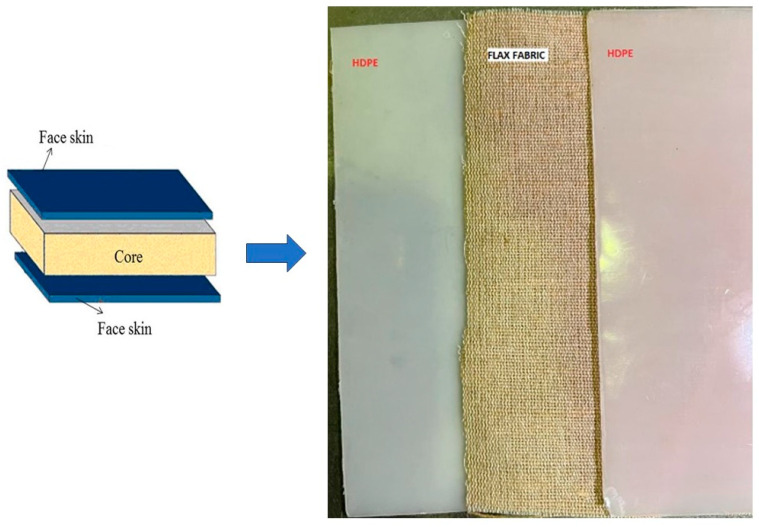
Laminated composite in which a unidirectional flax mat is sandwiched between two HDPE face sheets.

During the second step, a unidirectional flax mat measuring 160 mm × 160 mm × 1 mm was inserted between the HDPE face skins produced in step 1, as seen in Figure 1. The laminated material was thereafter subjected to compression molding at a temperature of 160 °C for a duration of 20 min under a pressure of 100 bars. A 20 s degassing phase was implemented midway through the operation to eliminate any trapped air bubbles. Afterwards, the material was rapidly cooled to a temperature of 30 °C over a time span of 10 min, resulting in the formation of a composite sandwich laminate measuring 160 mm × 160 mm × 2 mm. This composite plate was used to carve out tensile test coupons using a laser cutter, following the ASTM D 638 type IV [17] specifications.

### 2.3. Tensile Testing

Tensile specimens were prepared following the specifications of ASTM D638-22 for type IV samples. Five samples each from untreated and alkaline-treated groups were tested under quasi-static tensile-loading conditions utilizing a universal testing machine equipped with a 5 KN load cell. The velocity of the head during tensile testing was maintained at a constant value of 5 mm/min. Tensile modulus, tensile strength and strain to failure were recorded for all the tested samples.

### 2.4. Life Cycle Assessment

**Figure 2:**
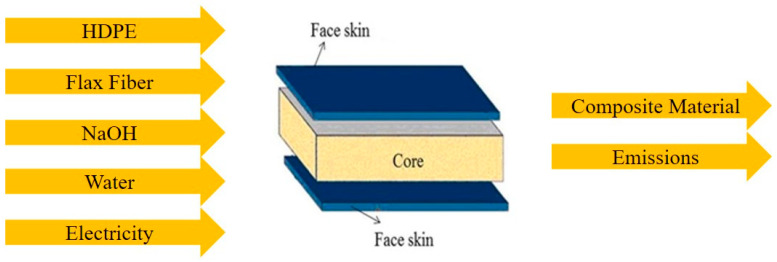
Input and output for LCA study and system boundary.

For this paper, LCA was conducted following the ISO 14040 standard which provides detailed steps to conduct LCAs. In accordance with this standard, there are four steps for an LCA study: goal and scope definition, compilation of inventory, environmental impact assessment, and interpretation of the results [16]. The first step in an LCA study is to define the goal and scope of study which includes delineating the system boundaries that are considered for the study and describing the functional unit for the study. System boundaries define the key elements of energy and material consumption that take place during the life cycle stages considered for the study. Additionally, using an appropriate functional unit for the study helps to provide a fair comparison between various alternatives considered in the study. The next step in an LCA study is to compile an inventory list. This list compiles the inflow of raw materials, energy, water, and outputs of the process. Next comes the environmental impact assessment due to the material and energy consumption. These impacts are reported in categories like global warming, acidification, and ozone depletion. In the final step of an LCA, the results of the study are analyzed to provide suggestions to reduce the environmental impacts. The current LCA study’s goal is to calculate the impacts of manufacturing flax fiber-based thermoplastics. For this study, a plate of 160 mm × 160 mm × 2 mm was used as the functional unit. All the flows are measured with respect to this functional unit. System boundaries for producing the material are shown in Figure 2. Materials, chemicals, water, and energy consumed for preparing flax fiber-reinforced plates are considered in the study. Earlier operations, like delivery of materials, packaging, and storage of materials, are not considered for this study. Since we are interested in comparing two types of processes for preparing these plates, we conducted a cradle-to-gate LCA study. Since we are interested in the process of producing biocomposites, we conducted a cradle-to-gate LCA. This LCA takes into account the life cycle from raw material extraction to the production of the product. The latter stages of transporting the material, use of material, and recycling of the material are not considered for this study. Table 1 lists the life cycle inventory (LCI) used in this study. It contains the quantities of material and energy used to prepare samples that are used as functional units for this study. To determine the environmental impacts, version 9.5.0.0 of SimaPro software was used, which is the leading software for conducting LCA and is widely employed in LCA studies published in reputed journals. To report the environmental impacts, ReCiPe MidPoint categories were used as ReCiPe is a widely used method for these types of assessments [16]. Midpoint categories report intermediate measures of environmental impacts that are caused by the material and energy use for a given product or a process. For example, CO_2_ equivalents can be used to indicate global warming potential of various gases that are emitted during the life cycle. In total, eighteen categories are reported to represent various impacts on air, water, land, and human health.

## 3. Results and Discussion

This section presents a detailed investigation of the mechanical properties and environmental effects of both untreated and alkaline-treated flax fiber-reinforced HDPE. The results are based on a comprehensive techno-environmental analysis.

### 3.1. Tensile Characterization

**Figure 3:**
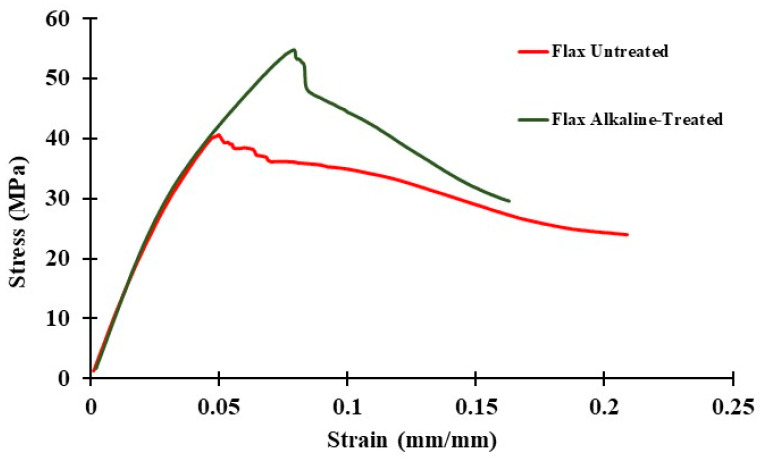
Representative stress-strain curve of untreated and alkaline-treated flax fiber-reinforced HDPE.

It has been estimated that the application of an alkaline treatment to flax fiber will result in an improvement in its mechanical properties by boosting the interfacial adhesion thereof. The stress–strain curve of untreated and alkaline-treated flax fiber-reinforced HDPE is depicted in Figure 3. This curve was acquired using uniaxial quasi-static tensile testing. The tensile strength of the composite material was greatly improved as a result of the alkaline treatment, as shown in Figure 3, which is overwhelmingly evident.

**Figure 4:**
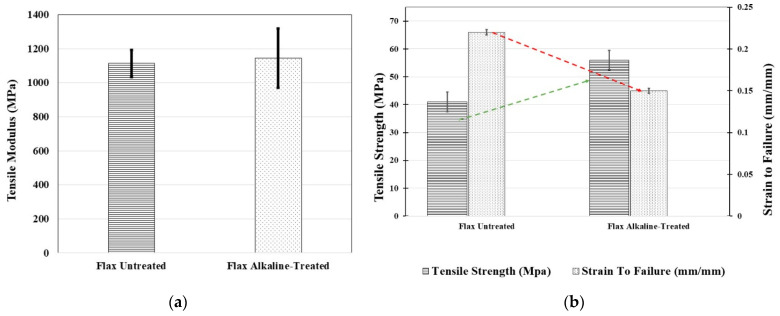
Tensile characteristics of untreated and alkaline-treated flax fiber-reinforced HDPE. (**a**) Tensile modulus; (**b**) tensile strength and strain to failure.

The quantitative changes of tensile modulus and tensile strength and strain to failure or ductility are depicted in Figure 4a,b, respectively. Despite the fact that there have been variations in the tensile strength and strain as a result of failure, the tensile modulus remained unchanged. While experiencing a forty percent decrease in strain to failure, the samples that were treated with alkaline showed a significant increase of 37 percent in tensile strength. It is possible to trace the change in tensile characteristics to the variation in interfacial properties that occurred between the fiber matrix and the alkaline treatment. A similar trend was observed in previous literature [18].

**Figure 5:**
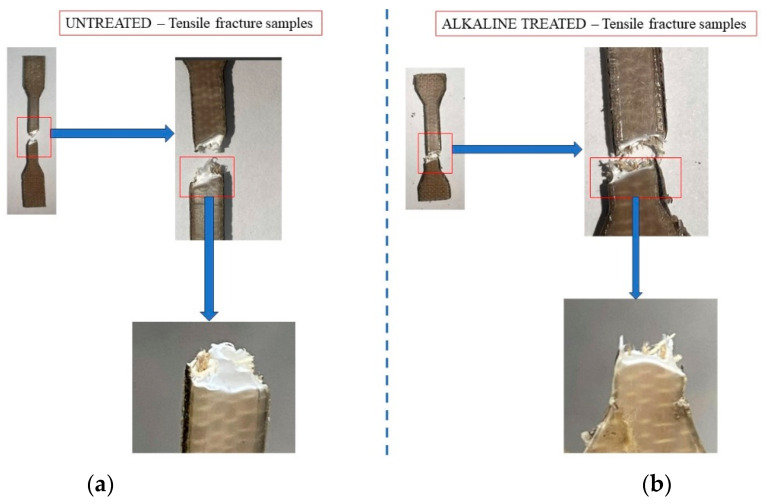
Tensile fracture surface of untreated and alkaline-treated flax fiber-reinforced HDPE. (**a**) Untreated; (**b**) alkaline-treated.

The physical and chemical changes may be evaluated on the fracture surfaces under tension (Figure 4), the surface profile of the fibers using SEM (Figure 4), and by Fourier transform infrared (FTIR) spectroscopy (Figure 5).

Figure 5 displays the surface of the flax-reinforced HDPE composites after undergoing alkaline treatment and without any treatment. The untreated samples displayed in Figure 5a exhibit noticeable slippage between the fibers and matrices, resulting in a ductile fracture of the matrix. Alkaline-treated samples showed improved adhesion between the fibers and matrices, resulting in a simultaneous and uniform failure of both fibers and matrices.

**Figure 6:**
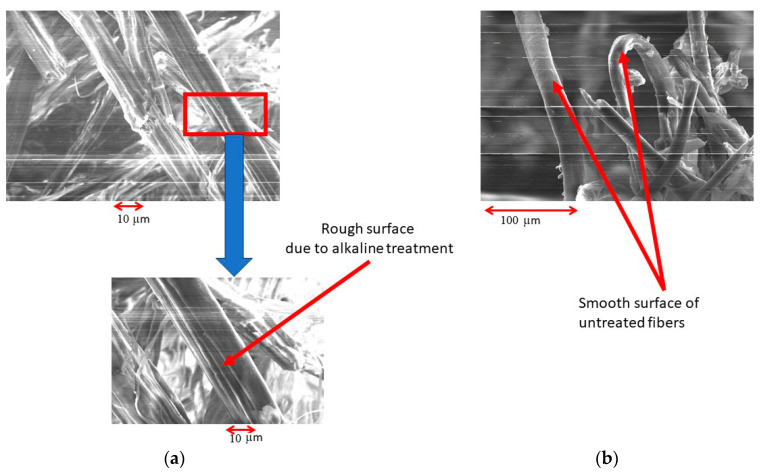
Scanning electron microscopy of untreated and alkaline-treated flax fiber. (**a**) Untreated; (**b**) alkaline-treated.

The impact of alkaline treatment on surface roughness was confirmed by examining the fracture surfaces using SEM, as seen in Figure 6. Alkaline-treated flax fibers display rough surface characteristics, unlike the untreated ones. On the surface of flax fibers, the SEM pictures reveal a sequence of longitudinal fissures. These cracks are an indication of the erosion of wax or the top layer of natural fibers. The adhesion of polymers can be improved by surfaces with a rough texture. In the case where it is construed as representing HDPE remnants, the argument will continue to be the same, given that HDPE residues are not discernible in the samples that have been treated. Owing to the complex material constituents of flax fibers, FTIR testing was detrimental to understanding the modified fiber due to alkaline treatment.

**Figure 7:**
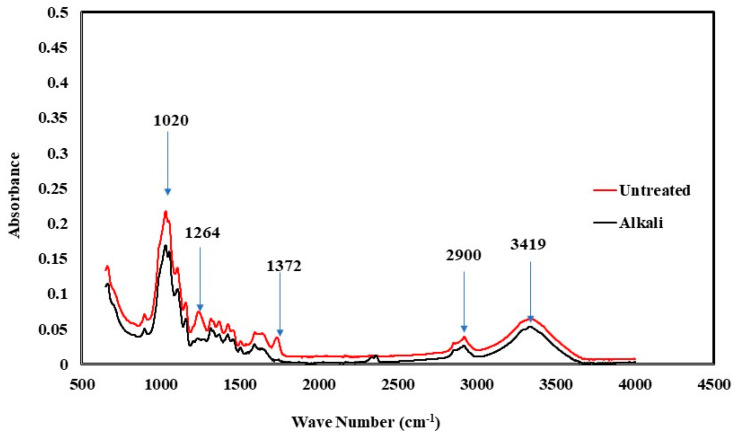
Fourier transform infrared spectroscopy of untreated and alkaline-treated flax fibers.

Figure 7 depicts the FTIR spectroscopy analysis performed on untreated and alkaline-treated flax fibers. Each signal observed in FTIR spectroscopy corresponds to a distinct chemical bond associated with a certain chemical composition, which in turn determines various thermo-mechanical properties. Untreated flax fiber typically comprises cellulose, hemicellulose, pectin, and lignin, along with four minor extractives, including wax, protein, fat, colors, and inorganic salts.

The samples treated with alkaline solution did not exhibit two distinct peaks, which are typically observed at around 1264 cm^−1^ and 1372 cm^−1^. The wave seen at 1264 cm^−1^ is indicative of vibrations that are characteristic of lignin. This outcome suggests a deficiency in lignin as well as some associated compounds, including wax and proteins. [13].

The wave at 1372 cm^−1^ corresponds to the asymmetric vibrations of lignin’s CH_3_ groups and the symmetric vibrations of its C-H groups. This observation further confirms the lack of lignin-related oscillations in the alkaline samples, which is distinctly evident when compared to the untreated samples [18,19]. Moreover, the SEM images confirm that the surface profile of flax fiber undergoes substantial alteration in alkaline samples, resulting in a rough surface. The FTIR data may be utilized to corroborate the surface roughness noticed in the SEM pictures, which can be attributed to the elimination of lignin, as well as the accompanying wax and protein.

### 3.2. Life Cycle Assessment

**Figure 8:**
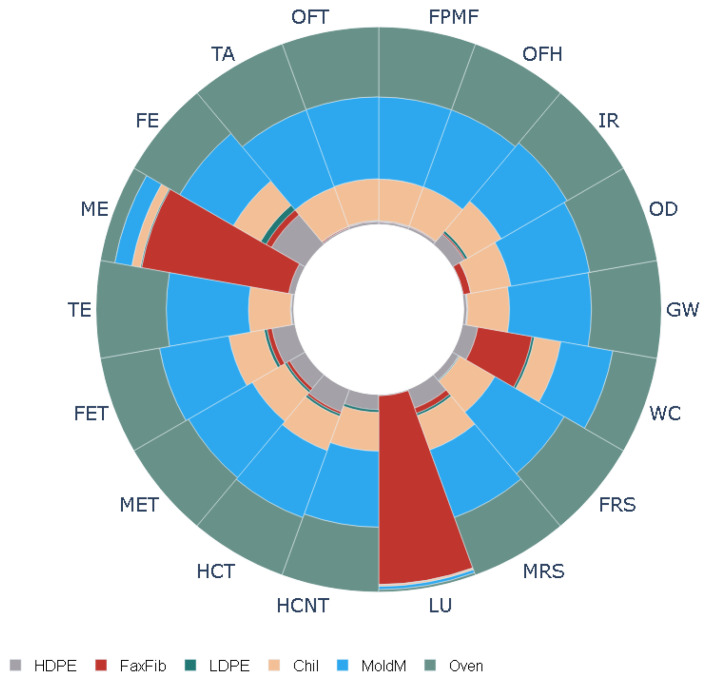
Normalized environmental impact categories for flax not treated with NaOH.

**Figure 9:**
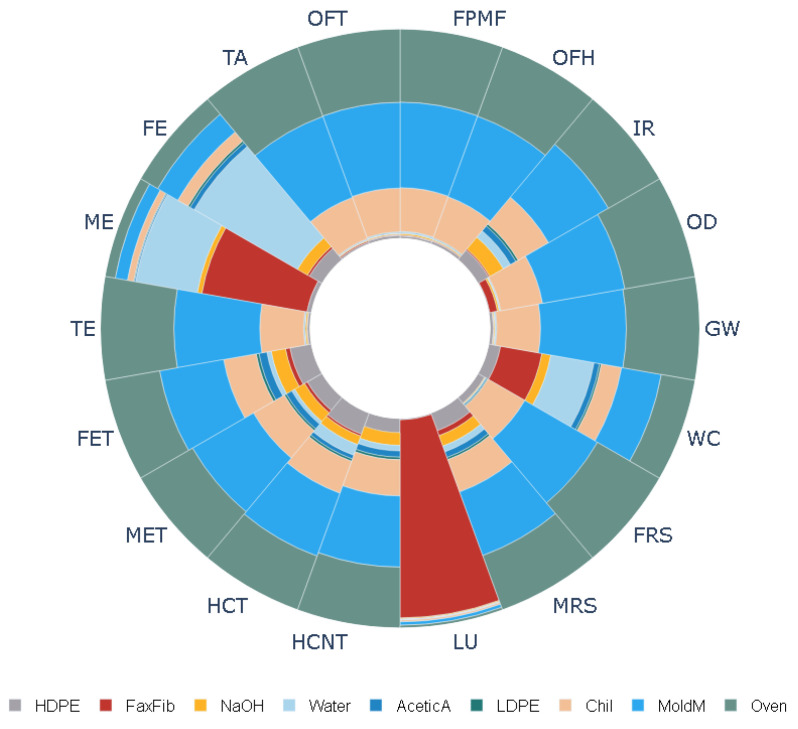
Normalized environmental impact categories for flax treated with NaOH.

The results of the LCA study are listed in Table 2. The table shows eighteen mid-point impacts using the ReCiPe impact assessment technique. The analysis was conducted for both untreated and treated flax fibers. The results show that the production of flax fiber composite plates treated with NaOH leads to a global warming potential (GWP) of 5.8 kg CO_2_, terrestrial acidification potential (TAP) of 0.0269 kg SO_2_, and human carcinogenic toxicity potential of 0.031 kg 1,4-DCB. Also, it can be seen from the table that treating flax fibers with NaOH increases the environmental impacts in all eighteen categories. For example, treating with NaOH gives rise to 18% more ionization radiation, 48% more marine eutrophication, and 38% more water consumption. Figure 8 and Figure 9 show the normalized values of energy and material consumption during the production of flax fiber plates. It can be seen that electricity is a major contributor to all the categories considered for this study (40% towards GWP, 41% towards TAP, etc.). Furthermore, flax fiber is a leading cause of the land use impact category (94%).

One reason for environmental impacts of production of flax fiber composite to be driven by electricity consumption is the type of electricity used in Saudi Arabia. The electricity used in Saudi Arabia is primarily generated by burning fossil fuels and this leads to global warming impacts as shown in the results. Using renewable energy during production of flax fibers can lead to reduced environmental impacts in nearly all the categories considered for this study. Using flax fiber also contributes to the land use category. This category takes into account the occupation or transformation of land for a particular use (in this case, growing flax plants). Land use can have direct and indirect impacts on several ecological parameters. For instance, land use can impact biodiversity, life support functions, and potential use of land in the future [20].

The findings of this LCA study point towards using renewable energy and optimizing land use for flax production to reduce the overall environmental impacts. Nevertheless, the gaps and limitations of this LCA study should be acknowledged. The inputs for the LCA study were primarily obtained from lab preparation of material samples. More accurate results can be obtained if the samples are prepared in an actual production facility. Similarly, the study was conducted in Saudi Arabia where LCA research is in the early stages. For this reason, reliable local LCI databases are not available. Use of a non-local database can lead to different impact values in LCA results. This LCA study can be further enhanced by addressing these limitations. Nevertheless, this LCA study provides valuable insight into the environmental hotspots present during production of flax fiber composites.

## 4. Conclusions

This study aimed to evaluate the mechanical characteristics, cost-effectiveness, and environmental impact of untreated and alkaline-treated flax-reinforced HDPE composites using a techno-environmental analysis. When flax fibers were subjected to an alkaline treatment, lignin, wax, and proteins connected to the process were removed, which resulted in a surface that was rougher. The tensile strength of the flax fibers was significantly improved as a result of this roughness, which strengthened their adherence to the HDPE. Both electricity and flax fibers were found to have contributed to the 18 environmental impact categories, as demonstrated by the findings of the life cycle study. This LCA contributes fresh information to the manufacturing of mesh membranes by coating, and it has the potential to assist in reducing the effect that the process has on the environment. Overall, this study can be used as a benchmark for the industrial implementation of alkaline treatment on flax fiber composites in mass production.

## Figures and Tables

**Table 1:** Life cycle inventory for preparing biocomposites.

Material/Process	Amount	Amount	SimaPro Material/Energy
	Without NaOH	With NaOH	
HDPE	0.042 kg	0.042 kg	HPolyethylene, high density, granulate {GLO}|market for|APOS, S
Flax fibers	0.007 kg	0.007 kg	Fiber, flax {GLO}|market for fiber, flax|APOS, S
Sodium Hydroxide	0	10 mL	Sodium hydroxide, without water, in 50% solution state {GLO}|market for|APOS, S
Distilled water	0	3190 mL	Water, ultrapure {RoW}|market for water, ultrapure|APOS, S
Acetic Acid	0	10 mL	Acetic acid, without water, in 98% solution state {GLO}|market for|APOS, S
Plastic bag LDPE	0.0049 kg	0.0049 kg	LPolyethylene, low density, granulate {GLO}|market for|APOS, S
Chiller	1.187 kWh	1.187 kWh	CElectricity, high voltage {SA}|market for|APOS, S
Molding machine	2.33 kWh	2.33 kWh	MMElectricity, high voltage {SA}|market for|APOS, S
Curing Oven	2 kWh	2 kWh	OElectricity, high voltage {SA}|market for|APOS, S

**Table 2:** LCA results.

Impact Category	Symbol	Unit	Impact Value	Impact Value	%Age Difference
			Without NaOH	With NaOH	
Global warming	GW	kg CO_2_ eq	5.745301679	5.811651	1.154844052
Stratospheric ozone depletion	OD	kg CFC-11 eq	3.60226 × 10^−6^	3.66 × 10^−6^	1.554498712
Ionizing radiation	IR	kBq Co-60 eq	0.036829709	0.043014	16.79235882
Ozone formation, human health	OFH	kg NO_x_ eq	0.014643984	0.014807	1.111626293
Fine particulate matter formation	FPMF	kg PM2.5 eq	0.008458484	0.008598	1.645062915
Ozone formation, terrestrial ecosystems	OFT	kg NO_x_ eq	0.014874519	0.015041	1.122139711
Terrestrial acidification	TA	kg SO_2_ eq	0.026735514	0.026978	0.907838854
Freshwater eutrophication	FE	kg P eq	9.44553 × 10^−5^	0.000267	182.495852
Marine eutrophication	ME	kg N eq	4.6778 × 10^−5^	6.94 × 10^−5^	48.34430937
Terrestrial ecotoxicity	TE	kg 1,4-DCB	14.77473319	14.98223	1.404388223
Freshwater ecotoxicity	FET	kg 1,4-DCB	0.02487353	0.028535	14.71901664
Marine ecotoxicity	MET	kg 1,4-DCB	0.042774812	0.047546	11.15483648
Human carcinogenic toxicity	HCT	kg 1,4-DCB	0.028180178	0.031875	13.11203442
Human non-carcinogenic toxicity	HCNT	kg 1,4-DCB	0.561270601	0.635217	13.17479091
Land use	LU	m^2^a crop eq	1.033990998	1.043837	0.952260482
Mineral resource scarcity	MRS	kg Cu eq	0.002061313	0.002317	12.40111921
Fossil resource scarcity	FRS	kg oil eq	1.866914949	1.889315	1.199826782
Water consumption	WC	m^3^	0.012643242	0.017524	38.6022898

## Data Availability

Data are contained within the article.
